# Continuous quality improvement as a tool to implement evidence-informed problem solving: experiences from the district and health facility level in Uganda

**DOI:** 10.1186/s12913-021-06061-8

**Published:** 2021-01-22

**Authors:** Hector Tibeihaho, Charles Nkolo, Robert Anguyo Onzima, Florence Ayebare, Dorcus Kiwanuka Henriksson

**Affiliations:** 1 Associate Child Fund International, Kampala, Uganda; 2 Liverpool School of Tropical Medicine, Liverpool, Uganda; 3 Makerere University College of Health Sciences, School of Public Health, Makerere, Uganda; 4 Karolinska Institute, Karoslinska, Sweden

**Keywords:** Continuous quality improvement, District health management team, Quality improvement, Uganda, Health facility

## Abstract

**Background:**

Continuous quality improvement processes in health care were developed for use at health facility level, and that is where they have been used the most, often addressing defined care processes. However, in different settings different factors have been important to support institutionalization. This study explores how continuous quality improvement processes were institutionalized at the district level and at the health facility level in Uganda.

**Methods:**

This qualitative study was carried out in seven districts in Uganda. Semi-structured interviews with key informants from the district health management teams and document review were conducted. Thematic analysis was used to analyze the data.

**Results:**

All districts that participated in the study formed Continuous Quality Improvement (CQI) teams both at the district level and at the health facilities. The district CQI teams comprised of members from different departments within the district health office. District level CQI teams were mandated to take the lead in addressing management gaps and follow up CQI activities at the health facility level. Acceptability of quality improvement processes by the district leadership was identified across districts as supporting the successful implementation of CQI. However, high turnover of staff at health facility level was also reported as a detrimental to the successful implementation of quality improvement processes. Also the district health management teams did not engage much in addressing their own roles using continuous quality improvement.

**Conclusion:**

The leadership and management provided by the district health management team was an important factor for the use of Continuous Quality Improvement principles within the district. The key roles of the district health team revolved around the institutionalisation of CQI at different levels of the health system, monitoring results of continuous quality improvement implementation, mobilising resources and health care delivery hence promoting the culture of quality, direct implementation of CQI, and creating an enabling environment for the lower-level health facilities to engage in CQI. High turnover of staff at health facility level was also reported as one of the challenges to the successful implementation of continuous quality improvement. The DHT did not engage much in addressing gaps in their own roles using continuous quality improvement.

**Supplementary Information:**

The online version contains supplementary material available at 10.1186/s12913-021-06061-8.

## Background

Poor quality of care is one of the reasons why mortality rates remain unacceptably high in many Sub-Saharan African (SSA) countries like Uganda [1–3] despite the existence of effective interventions that could significantly reduce mortality [4–6]. Approximately 500,000 stillbirths and 1 million newborn deaths could be prevented if the quality of care was improved [4]. In many SSA countries like Uganda, health care delivery is decentralised to the district level, making the district health system responsible for the quality of care that is provided [7, 8].

Quality improvement (QI) efforts have become common within healthcare [9] with several approaches used, including Continuous Quality Improvement (CQI) processes. CQI is a quality management process that encourages all health care team members to continuously ask the questions, “How are we doing?” and “Can we do it better?” [10] with improving organisational processes by applying scientific methods being key to a better quality. One of CQI’s important contributions is its development of effective, simplified techniques for applying scientific approaches to the improvement of daily work processes [11]. For healthcare, the goal of CQI is to consistently meet or exceed the needs of caregivers, patients, service providers and the community. CQI has mostly been used at the health facility level, often addressing defined care processes [6]. In decentralized health systems like in Uganda where the District Health Management Teams (DHMT) have the overall responsibility for the quality of care provided, it is important to understand their role in implementation of CQI processes. Insights from the CODES project that introduced CQI processes at the district level can contribute to understanding the institutionalization of CQI processes at the district level. Institutionalization refers to ‘the action of establishing something as a convention or norm in an organization or culture.’

### Continuous quality improvement in the CODES project

The Community and District Empowerment for Scale-up (CODES) project in Uganda adopted continuous quality improvement processes, at district and health facility level as one of the approaches to build the capacity of health managers and health workers alike to utilise locally-generated evidence and solutions to address performance gaps in the delivery of quality child survival interventions [12, 13]. The CODES project utilized CQI to improve managerial problem-solving and quality of care at the points of service delivery. The project trained district health management team (DHMT) members to utilize CQI processes, who in turn trained, mentored and supervised health facility staff in the implementation of CQI processes [13]. The CQI principles used in the CODES project included 1) training managers to understand and accept the long-term commitment to pursuing quality improvement, learning from best practices and in turn sharing experiences, 2) a continuous effort to improve the process, with numerous small improvements, 3) empowering managers by giving not only the authority but also the training and the resources necessary to improve the process, 4) use of data to manage and improve the process, and 5) top management’s commitment.

**Fig. 1:**
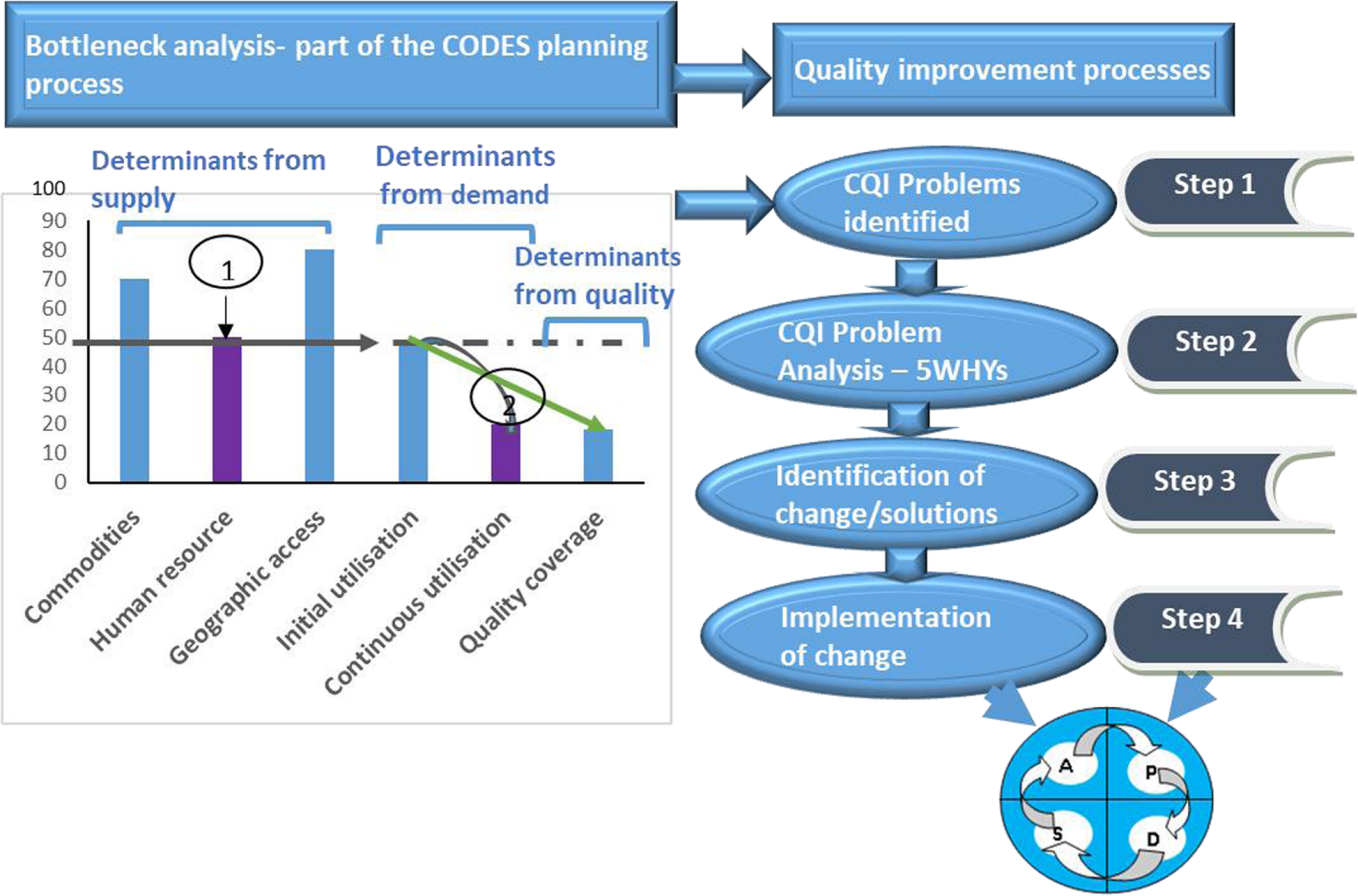
Shows the Tanahashi model that was used to identify health system bottlenecks and the steps of the Continuous quality improvement process used in the CODES project

In line with the national quality improvement framework, the CODES project introduced CQI processes to 13 project implementation districts. CQI was introduced in two phases; the first phase which was the proof of concept period referred to as Wave 0 and the second phase which was the trial period referred to as Wave 1 [12, 13]. See Fig. 1.

During the CODES project, CQI processes by the DHMT members were initiated right from the planning process [12, 13]. The approach taken to support CQI in the districts was through training and mentorship at district and health facility level. The DHMT members were trained and supported by the project team to train health facility workers to enable them to identify and solve problems using CQI techniques. Also, the DHMT members mentored health facility staff in the implementation and monitoring of CQI processes at the facility level.

Each district health management team identified their so-called bottlenecks, (that were also referred to as problems) to service delivery of child survival interventions using the modified Tanahashi model of bottleneck analysis [13–16], as shown in Fig. 1. During the bottleneck analysis process, problems requiring CQI processes were identified for action at either DHMT level or at the health facility level. The DHMT members communicated the identified problems that required health facility level action to health facilities and supported health facility staff to implement CQI processes to overcome the problems or bottlenecks. The DHMT also initiated their CQI processes for problems requiring DHMT-level action. CQI processes, as shown in Fig. 1, were then applied to determine the causes of the bottlenecks and identify solutions [13–15].

Typically, identified solutions were categorised into two; The first category were solutions that could be addressed at the level of identification for example at the facility or district level using available resources at that level. Such solutions included, for example, instituting triage of patients at OPD to improve patient flow. The second category of solutions were those requiring interventions from a higher level, for example, the district local government, the ministry of health or implementing partners. Solutions like costly capacity building training fell into this category. Identified solutions were implemented and monitored utilising the Plan-Do-Study-Act (PDSA) cycles.

Continuous quality improvement has been used with reasonable success in Ethiopia to build *Woreda* (district) leadership capacity to facilitate continuous improvement of maternal, neonatal and child health at the community level [17]. In Rwanda, nurse mentors were trained in CQI and mentorship techniques and integrated into the Ministry of Health (MoH) district supervision team, which resulted in a significant improvement in several quality-of-care indicators [18]. A study in Kenya also showed that training supervisors to improve reproductive health resulted in significant improvement in the quality of health care for the supervisors, the providers, as well as client-provider interactions [19]. Different factors have been important in the different countries to support institutionalization of Quality improvement processes.

In Uganda, the ministry of health established the first Quality improvement program, the Quality Assurance Program (QAP) in 1994 [20]. The program which was aimed at strengthening district-level management of primary health care services recorded considerable gains in a relatively short time [20]. Quality Improvement interventions were subsequently mandated by the Health Sector Strategic and Investment Plan (HSSIP) [21] and the current Health Sector Development Plan (HSDP) [22]. The national QI strategies are operationalised in the Health Sector Quality Improvement Framework and Strategic Plan (QIF&SP) [23]. Although significant progress has been made, substantial challenges have been reported which included weak mechanisms to coordinate QI initiatives at all levels, lack of resources, and managerial capacity at the district level [20, 24]. The CODES project aimed at addressing the managerial capacity gap through building managerial capacity and supporting district managers’ engagement in CQI processes. Therefore this study was conducted in some districts that were implementing the CODES project to understand the institutionalization of CQI processes at the district level.

The objective of this study was to understand how the continuous quality improvement processes introduced by the CODES project were institutionalized at the district level.

## Methodology

### Study design

A qualitative research design was employed because it allowed for a better understanding of the implementation of CQI processes at the district and health facility level [25, 26]. District documents relevant to the CQI process were also reviewed.

### Study sites and selection criteria

This study was conducted in seven purposively selected districts from 13 districts that were implementing the Community and District Empowerment for Scale-up in Uganda described elsewhere [12, 13]. The seven districts in this study were selected to represent districts that had been implementing the CQI process for three years (wave 0) and two years (wave 1). Districts were also selected depending on their year of establishment. If they were established before 2010, they were referred to as ‘old districts’ and if they were in 2010 and after they were referred to as ‘new districts’ as shown in Table 1.

**Table 1:** Districts included in the study

Years of implementation of CQI	Old districts	New districts	Total
Three years (Wave 0)	Masaka, Wakiso Mukono	Bukomansimbi	4
Two years (Wave 1)	Apac	Buhweju, Maracha	3
Total	**4**	**3**	**7**

It was assumed that including districts that had been implementing the CQI processes for varying periods could likely show differences if any that could result from length of exposure to the CQI processes. Similarly, districts that were established within different periods could show differences due to characteristics that may result from being ‘old’ or ‘new’ districts. The selected districts are in central (Bukomansimbi, Masaka, Mukono and Wakiso), Western (Buhweju) and Northern (Maracha and Apac) regions of Uganda.

**Fig. 2:**
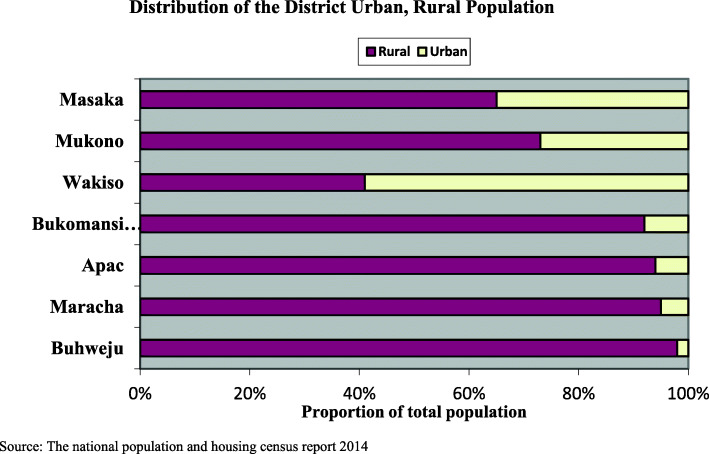
Shows the rural and urban population distribution in all the study districts in the CODES project. Source: The national population and housing census report 2014

According to the 2014 Uganda national population and housing census report [27], the study districts had the majority of the population living in rural areas; Buhweju (98%), Maracha (95%), Apac (94%), Bukomansimbi (92%) and Masaka (65%), Mukono (73%) and, Wakiso (61%) as shown in Fig. 2.

### Study participants and sample size

Study participants were purposively selected because they participated in CQI implementation [28] and their availability and willingness to participate in the study Each of the participants had one of the following roles within the CQI process, a CQI district focal person, a member of the District Quality Improvement Team (DQIT) or the district focal person for the CODES project. The participants also had the following designated roles as members of the district health management team, District Health Officer (DHO), district biostatistician, district nursing officer, district CQI focal persons (a role delegated by the DHO to a member of the DHMT or health facility). The study assumed that given their involvement in the CQI process, they would provide relevant information. A total of 15 interviews were conducted. One participant from Wakiso district, one from Masaka, two from Mukono, two from Bukomansimbi, three from Apac, two from Buhweju, and four from Maracha district. We had planned to interview at least 14 respondents, at least 2 from each district. However, two of the respondents (from Masaka and Wakiso districts) were not available to participate in the study as arranged. The interviews were stopped when no more new information was obtained.

### Recruitment of participants and consent

Participants were invited to take part in the study through the District Health Officer (DHO). After the participants agreed to take part, telephone calls were made to each of the participants and appointments were made for the face - to - face interviews. More information about the study was given, which included the fact that they were not getting paid for the interviews, and their participation was voluntary. Individual verbal informed consent was obtained from the participants at the beginning of each interview. The interviewers had prior interaction with some of the study participants during implementation of the CODES project.

### Data collection procedures

Data were collected between March and April 2016. Semi-structured interviews were conducted in English since all the participants were fluent in English. Interviews were conducted either at the respective district health offices or during a CODES project workshop. Each interview was conducted in privacy with only the interviewer and respondent able to hear the proceedings. During the interviews, participants were asked questions related to leadership and governance, CQI culture, structure and functions, and support to quality assurance. Probing was done to get a deeper understanding of the CQI processes and experiences [29, 30]. Interviews were audio-recorded and lasted approximately 60 min. All interviews were transcribed verbatim. Document review was also conducted on CODES project CQI annual reports from each of the districts and annual comprehensive district reports. No repeat interviews were carried out and transcripts were not returned to the respondents for correction.

### Interview guide

Development of the interview guide, as shown in Additional file 1, was informed by the organisational aspects of the Model for Understanding Success in Quality (MUSIQ) [9]. The MUSIQ model was chosen to inform the development of the interview guide, as shown in Additional file 1, because it provides a lens through which the implementation of QI programs can be studied at an organizational level, at the level of the quality improvement team and also at the micro level. The MUSIQ model also shows how context influences the success of individual quality improvement projects. The interview guide, as shown in Additional file 1, was developed around the district health management team and the quality improvement team levels of the health system, and within these levels contextual factors related to leadership and governance, QI support and capacity and characteristics of the QI team.

### Study team

The research team consisted of a public health specialist and health systems specialist (DKH) with experience as head of a district health system, a public health specialist and trainer of quality improvement in health (HT), a health systems/services management specialist (RA), a statistician (CN) with experience in public health and a social scientist (FA). No one in this team was working within the district health system by the time of CODES project implementation.

### Data analysis

**Fig. 3:**
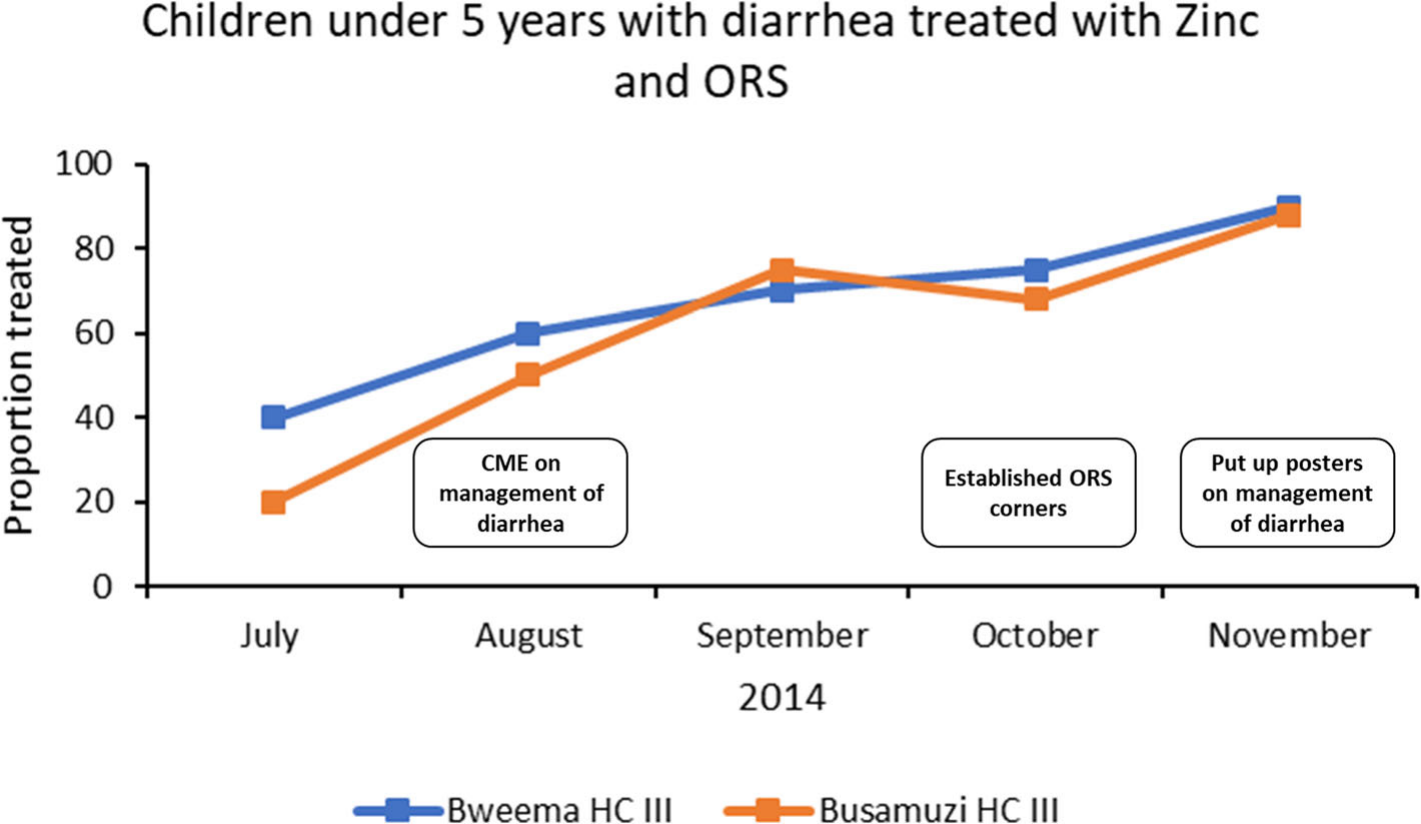
Shows the proportion of children under five years with diarrhea who were treated with Zinc and ORS between July and November 2014 at two health facilities. The figure also shows the quality improvement actions during the same period at each of the health facilities

**Fig. 4:**
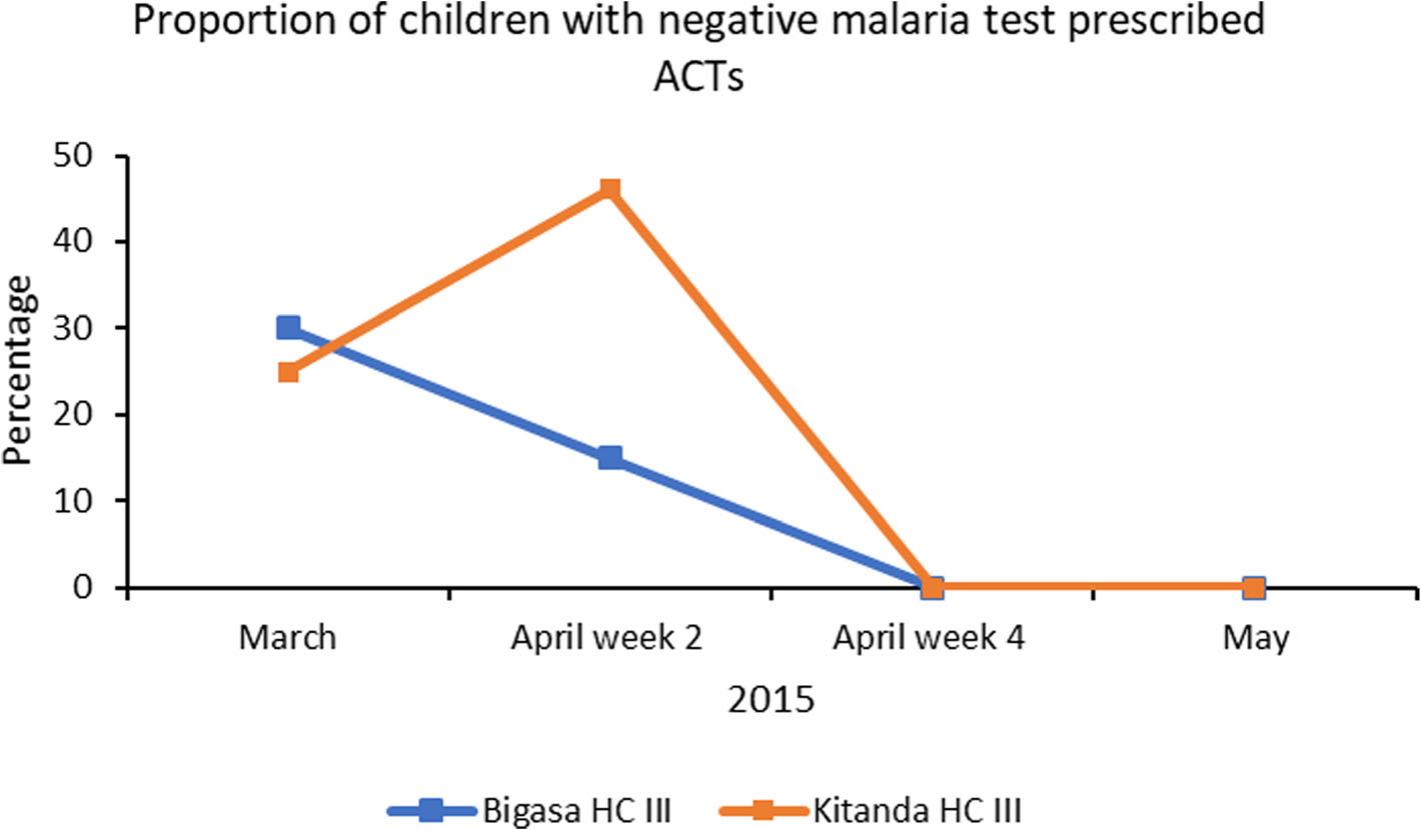
Shows the reduction in the proportion of children with negative malaria test results prescribed anti-malarial treatment after implementation of quality improvement processes

Four of the authors (RA, CN, HT and DKH) independently read through three transcripts. They then came together to discuss and establish consensus on the coding of the data. This process established a common meaning of the data. After an agreement had been reached on the coding process, each of the authors analysed data from three or four transcripts. A deductive process of thematic analysis was used to classify data into themes [31] that were informed by the contextual factors from the MUSIQ model. Some of the data collected was not represented in the contextual factors from the MUSIQ model and some of the data was allocated to more than one of the contextual factors. Results were therefore reported according to the themes that were derived from the data. The document review on CODES project CQI annual reports from each of the districts and annual comprehensive district reports enabled triangulation of findings from the interviews and was used to identify examples of quality improvement processes as illustrated in Figs. 3 and 4 in the results section.

## Results

### Structure, functions and culture of continuous quality improvement teams

Overall, district health managers reported significant improvement in the culture of quality improvement through acceptability of CQI principles and formation of CQI teams both at the district and health facility level. All districts that participated in the study formed CQI teams both at the district level and at the health facilities. The district CQI teams comprised of members from different departments within the district health office under the leadership of the DHO who provided oversight to the team. Composition of the CQI teams varied between districts with a membership of between 7 and 9 people. Membership included, for example, the biostatistician, Maternal and Child Health Officer (MCH), environmental health officer, officers from TB and malaria control, and laboratory personnel.

District level CQI teams were mandated to take the lead in addressing management gaps and follow up CQI activities at the health facility level. Monitoring of CQI activities at the health facility level was done during regular support supervision visits. DHMT members and health workers were provided with the knowledge and skills about CQI principles through the training and mentorship that was provided by the CODES project. Which knowledge and skills resulted in better management of clients, as mentioned in this quote.

*“Everybody knows that really the client is the boss so unlike those days when people used to be rude you know A, B, C, D. I think CQI has been the turning point for most of our health workers now.”* **(CQI team member, Mukono)**

Quality improvement activities were implemented with a focus on district and health facility-specific gaps identified by the CQI teams. Examples of CQI activities included keeping offices clean, improving record-keeping and data reporting through the Health Management Information System (HMIS). One of the district managers had this to say.

*“As a district, we sought to improve on the reporting, timeliness and then quality of data by ensuring that all health facilities submitted their monthly reports by 7th every month. The Biostatistician was tasked to monitor this indicator*.” **(DHT Member, Mukono district)**

In addition to data quality, districts also reported the identification of gaps in areas of support supervision. As such districts were able to mobilise for resources to enable regular support supervision.

*“ … … … thanks to CQI initiatives irregular support supervision to the lower-level facilities is a thing of the past.”* **(DHT member, Maracha district)**

At the health facility level, health workers were able to hold regular review meetings to identify gaps in service delivery, their causes and developed action plans to address the gaps within the CQI priorities of the district.

Majority of the respondents also reported that CQI principles improved service delivery, for example, better triaging to ensure that emergency cases were handled urgently and reducing the waiting time at the outpatient department. Also, the attitude of the health workers has changed with focus on the needs of the clients prioritised during service provision. Some DHMT members were aware of existing reporting mechanisms for CQI activities at the health facility level through the health management information system as elaborated by the following quotes:

*“ … … .at the facility level, quality improvement teams conduct monthly meetings. They identify the gaps that hinder their performance, and they take one by one and set targets on how to improve … .”* **(DHT member, Maracha district)**

*“ … … . there are existing Quality Assurance guidelines from the Ministry of health, especially for health facilities. All health facilities must report on quality improvement activities every month.”* **(DHT member, Bukomansimbi district)**

CQI principles were also reported to improve teamwork with DHMT members taking their responsibilities seriously, as partly elaborated in this quote.

*“ … … talking of values, one is teamwork, at least we’ve tried to work together as a team. We identify problems, identify indicators together and share responsibilities.”* **(DHT member, Buhweju district)**

### Leadership, governance and support for quality improvement

Acceptability of CQI by the district leadership was identified across districts as supporting the successful implementation of CQI. Involvement of district leadership outside the health department, for example, the Chief administrative officer was key for the district ‘buy-in’ and implementation at the health facility level.

*“And then support from our political leaders, they have good support towards our programs and they are always close to us at every level, they are very committed and very supportive that’s what facilitated the improvement in the, in the CQI”.* **(DHT member, Apac district)**

Support for CQI processes was reportedly evident, after training, mentorship and support to the DHMT who in turn supported health facility teams during regular supportive supervision visits. The support provided reportedly led to a ‘new’ way of problem-solving, and a more systematic and multidisciplinary team approach. As mentioned in this quote.

*“ … . CQI has made a lot of improvement (to support supervision) because the members (of the support supervision team) are drawn from different departments … . when we go together, we come up with a holistic approach to the problem. We think that CQI helps us to solve several problems at once … .”* **(DHT member, Maracha district)**

In other districts, the knowledge and skills acquired in CQI made the DHMT to broaden the scope of CQI given that the DHMT had the liberty to prioritise CQI at that level compared to earlier CQI initiatives that focused on improving donor priorities as one respondent had this to say.

*“CQI by the time I just entered service around 2010, was under HIV, care initiatives. They visited us all the way from Kampala once in three months. We didn’t understand the full aspect of quality improvement until the CODES project training. We now fully understood the components of quality improvement apart from the HIV quality of care. It allowed us to incorporate all other aspects of health care including human resource management. … ”.* **(DHT member, Buhweju district)**

There were reported variations in CQI uptake at the health facility level. The variation was attributed to several factors including the commitment of leaders at this level, specifically health facility in charges. Respondents reported that in health facilities with committed in charges (leaders), CQI uptake was more successful.

High turnover of staff at health facility level was also reported as a detriment to the successful implementation of continuous quality improvement. In some cases, health workers who had received CQI training had either been transferred or left employment. This affected implementation of the principles as newly recruited staff would require training before they could contribute meaningfully to the CQI activities at the facilities.

*“ … for example at facility level you get a team, you train them, they start implementing these activities (CQI) and then they (person) is transferred or drops out and you have to pick another person and, train them. Throughout the turnover of staff was one of the challenges … ”.* **(DHT member, Bukomansimbi district)**

Across all the districts, DHMT members were aware of existing policy guidelines on quality assurance from the Ministry of Health. Although district managers reported that quality improvement interventions were guided by MoH’s quality assurance guidelines, the majority were unable to elaborate on the guidelines.

As a result of documented benefits from CQI activities funded by UNICEF, DHMTs in some districts were able to request and receive additional funding for CQI activities from other stakeholders as stated by one of the district managers;

*“We have been able to convince other partners to help fund some of the CQI activities which were not earlier being funded.”* **(DHE, Buhweju district)**

District managers acknowledged that before the CODES project, they lacked the knowledge and skills to use of CQI principles. The CODES project provided training and mentorship to DHMTs. The district managers, in turn, trained and provided mentorship for health works at the facility level. As a result of the capacity building, district managers were able to use their context-specific data for quality improvement. Some of the district managers had this to say.

*“ … … .. am glad to say that as a DHMT we were privileged to be trained under this project in quality improvement, with funding obtained from the same project we have been able to cascade CQI in all facilities. Initially CODES helped us train in four facilities but again with further funding we continued and trained in all facilities … … .”* **(DHO, Maracha district)**

*“We can now use our routine data, identify problems and develop solutions using PDSA; … ..possibly with funding, we can conduct LQAS survey as a district to generate our data.”* **(CQI focal person, Apac district)**

Figures 3 and 4 show examples of how continuous quality improvement practices improved the quality of care at four health facilities. Improvements that were made for the management of diarrhea with Zinc and ORS (Fig. 3) included 1) CME sessions on management of diarrhea 2) establishment of ORS corners and 3) pinning up posters to remind clinicians to prescribe Zinc and ORS for diarrhea cases.

Figure 4 shows a reduction to zero of children who tested negative for malaria receiving anti-malarial treatment. Reduction was achieved through training of health facility staff in CQI, sharing district CQI priorities with health facilities during the CQI training, supporting health facilities to form CQI teams, mentoring and support supervision, and organising inter-health facility CQI collaborative learning meeting.

## Discussion

The results revealed that support for continuous quality improvement principles started with buy-in from the district health management team members who in turn supported lower health facilities to integrate CQI in their work. Although acceptance of the continuous quality improvement principles in some health facilities was less than ideal, in most health facilities, there were positive benefits. CQI reportedly *flourished* in the districts partly because of the enabling environment for integration of CQI principles into the routine workings of the health system. District managers reported that quality improvement interventions were guided by MoH’s quality assurance guidelines. However, the majority of DHMT members were unable to elaborate on the guidelines.

The study demonstrated that with requisite training and skills, district health managers could facilitate the adoption of CQI principles at district and health facility level. Adoption of CQI was facillitated by the district managers´ engagement in institutionalisation of CQI, district-level direct implementation of CQI and enabling of CQI at health facilities.

Continuous quality improvement was institutionalised through the formation of district quality improvement teams and supporting the formation of the same at health facilities. The teams consist of members of the DHMT and health workers with different specialities and technical abilities. The multidisciplinary team is consistent with the MoH’s national quality improvement manual that recommends the formation of multidisciplinary health facility teams to cultivate the quality improvement culture at the health facility [32].

Our study demonstrated that having a multi-disciplinary team was one of the contextual factors for successful implementation of CQI, as was suggested by Kaplan et al. and Øvretveit J [9, 33]. Through this structure, the managers were able to continuously identify and discuss quality-related issues and provide systematic support (training, supervision and mentorship) to the health workers. Implementation of CQI at health facility level was better supported when health workers were equipped with problem identification and solving skills.

District health management teams play an important role in supporting health facilities to adopt and integrate CQI principles in their routine operations.

Once equipped with knowledge and skills, front line health workers can identify performance gaps which hinder the delivery of quality health services as well as design local solutions to bridge these gaps. This support not only equips the health workers with technical knowledge and skills but also motivates operational-level health workers. Through this study, health workers reported improvements in service delivery by holding regular meetings at the health facilities and following up on identified solutions to ensure that action was taken. Health workers reported seeing improvements in performance, specifically, reduction in waiting times at outpatient departments, reduction in absenteeism and late coming. Also, health workers reported improvements in clinical practice ensuring orderliness in triaging and dealing with emergency cases urgently. Similarly ensuring that prescriptions are only issued upon laboratory confirmation of disease, thus providing safe and quality care and reducing wastage of medicines.

Many of the DHMT members were directly engaged in CQI implementation. However, almost all the outputs for the DHMT-implemented CQI projects like improving absenteeism, quality of reports among others were tagged to health facilities. This is not surprising given that many districts placed adherence to quality improvement principles – with ‘client focus’ as one of such principles [34]. This finding implies that the client in the perspective of DHT is the patient or client who is found at the health facility. This perception leaves the DHMT with no CQI projects directly related to their roles for which the client would be alternative (not necessarily a patient/client). For example, a DHMT that has gaps in performance management could focus CQI on that role that exhibits the need for improvement, and the client could turn out to be an operational-level health worker.

District managers also played an important role in creating a quality improvement culture with health facilities forming CQI teams that meet regularly to review the progress of identified gaps and proposed solutions to address them. Health workers have, as a result, integrated CQI into their routine operations of the health facility and have registered significant changes as a result of CQI adoption.

Challenges like inadequate funding, poor-commitment of lower-level leadership and high turn-over of staff regress the implementation and outcome of CQI by the DHMT. In this study, we note the limited commitment of the government of Uganda to funding CQI. However, some DHMTs committed the entirety of the project ‘slush’ fund to quality improvement. Also, CQI provided a platform for mobilisation of resources from development partners. The provision of adequate resources in quality and quantity is a long-term adjustment managers must make to ensure the provision of good care. This implies that the DHMT members in the districts may face a stiffer challenge with CQI implementation if no additional funding is provided. The MoH must make a deliberate effort to commit resources to CQI on annual bases since the benefits of CQI at this level are tangible.

### Methodological considerations

The study was conducted in only seven districts and therefore limits the generalizability of findings. However, the districts in the study are therefore a fair representation of many districts in Uganda, although the specific context will vary. There was also uneven distribution of participants across districts due to availability of DHMT members at the time of data collection, which also limits generalizability. The study did not collect any information from the health facility level where most of the implementation of quality improvement processes took place and would therefore have an effect on institutionalization of CQI processes. This study is exploratory and does not address causality between study interventions and outcomes. Even with these limitations, the study provides insight into the institutionalization of CQI processes at the district level.

## Conclusion

The study identified the leadership and management provided by the district health management team as an important factor for the use of CQI principles at the district and health facility level. The key roles of the DHT revolved around the institutionalisation of CQI at different levels of the health system, monitoring results of CQI implementation, mobilising resources and health care delivery hence promoting the culture of quality, direct implementation of CQI, and creating an enabling environment for the lower-level health facilities to engage in CQI. DHMT’s as direct implementers of quality improvement had little on quality improvement processes directly addressing their roles as managers,. Future planners and implementers of CQI at district-level should also engage CQI processes that directly address gaps related to their roles as district health managers.

## Supplementary Information

**Additional file 1:.**

## Abbreviations

CQI: Continuous Quality Improvement
QI: Quality Improvement
PDSA: Plan-Do-Study-Act
DHT: District Health Team
DHMT: District Health Management Team
DHO: District Health Officer
DHE: District Health Educator
MoH: Ministry of Health
CODES: Community and District Empowerment for Scale-up
HMIS: Health Management Information Systems
MUSIQ: Model for Understanding Success in Quality
UNCST: Uganda National council of Science and Technology
ACT: Artemisinin-based Combination Therapy
