# Supplementary material for: Continuous quality improvement as a tool to implement evidence-informed problem solving: experiences from the district and health facility level in Uganda
Source: BMC Health Serv Res. 2021 Jan 22;21:83. doi: 10.1186/s12913-021-06061-8 (PMC7825214; doi:10.1186/s12913-021-06061-8)
Supplement: Supplementary file 1 — Additional file 1:. [file 12913_2021_6061_MOESM1_ESM.doc]

**Date interview conducted: .................................................................**

**District: ........................................................................... Wave: ..................................**

**Section I: Characteristics of the respondent**

**Title of the respondent**: ......................................................., **Age:** ......................................

**Sex**: ...................................................................................

**Note: Begin every interview with recorded introduction (including title[s]) because this will be important in transcription.**

**Section II: Starting questions for assessing implementation of CQI at district level**

Leadership and governance-related issues

1. Policy: Is there policy on Quality assurance available at this district? Are there policy guidelines that support or reinforce quality assurance or quality improvement at the district?
2. Describe priorities you have set in the past as a leader that put QA/CQI above other areas of health care service provision.
3. What are the core district health office values you would say emphasize continuous quality improvement (CQI)?
4. Describe resources – personnel, funds, time (absolute amounts and proportions) your office has allocated to QI activities in the past 1 year? What is the estimated proportion of these resources that were from the government and what is the proportion of donor funded resources?

CQI culture, structures and functions

1. Does the district have a CQI team? If yes, please describe the composition including leadership and functions of each of the members.
2. Please describe to me the culture of Quality assurance and CQI in this district in the past 5 years?
3. Describe the (QA) technical capacities that exist among each of the CQI team members (Training, skills, and/or experience).

Are there activities of CQI at DHT level that take place in this district? Since when did (your) DHT start implementing CQI?

1. How does DHT identify CQI projects? (Probe if BNA/CA contributes to identifying CQI problems/interventions. In addition, ask for other ways that guide them to identify the problems). After identifying problems that require DHT-level CQI, how does the DHT proceed (probe for processes after problem identification: e.g. problem analysis, identification and implementation of solutions; and the roles of CQI members in these processes) **PROBE: How were principles of CQI applied**.
2. What CQI interventions have been completed by the DHT? What are the current CQI interventions the DHT is carrying out?
3. Relating to the CQI projects (if any) in (5) above, how has CQI contributed to improving on the functions and activities of the DHT? (Probe specific CQI projects that contributed to the improvements). Do you think the DHT-level CQI has contributed to improving service delivery at health facilities? If yes how?
4. What went well during the CQI implementation? What influenced the processes that went well (probe at policy/guidelines, capacity, motivation, resources etc)
5. What were the strengths of the DHT in accomplishing the various CQI projects?
6. Share with us the motivation and challenges during implementation of CQI at DHT level (probe at policy/guidelines, capacity, motivation, resources etc).
7. How best could CQI at DHT level be improved?
8. Would you continue implementing CQI at DHT-level or recommend its roll-out to other districts (give reason[s] for your answer)

Support to QA

1. What has the DHT done to build capacity at district and health facilities in CQI in the past 1 year? What specific roles did the DHT have in these capacity building activities?
2. How do you share information on and communicate CQI related activities with stakeholders (health facilities and other partners)? (Probe in relation to what was done in the past 1 year). Describe when and how your office has rewarded or recognised individuals or teams that have performed well towards QA and QI.
3. What kind of documentation exists in this district for QA/CQI activities?
